# Experimental model for percutaneous tracheostomy training^☆^^☆☆^

**DOI:** 10.1016/j.bjorl.2025.101612

**Published:** 2025-05-27

**Authors:** Caroline Schmiele Namur, Kassem Samir Saleh, Marianne Yumi Nakai, Alexandre Baba Suehara, Antonio Jose Gonçalves

**Affiliations:** Faculdade de Ciências Médicas da Santa Casa de São Paulo (FCMSCSP), São Paulo, SP, Brazil

**Keywords:** Tracheostomy, Training, Learning

## Abstract

**Objective:**

To propose a practical and accessible model that enables the training of this technique.

**Methods:**

The model comprises a structure composed of a wood board resting on a PVC tube. A foam block on which the pig trachea is supported is placed on this structure. A foam sheet covered with fabric is placed over the trachea to simulate the soft parts of the neck and the skin. This is a practical training model for practice of surgeons in percutaneous tracheostomy. A questionnaire on the assessment of the proposed model to each participating surgeon was applied.

**Results:**

Ten surgeons underwent training on the model: two experienced head and neck surgeons, three specialty residents, one otorhinolaryngologist, and three general surgery residents. All of them were able to perform the procedure successfully.

**Conclusion:**

The proposed simulation model presented good results when applied to surgeons, and it is a possible alternative to percutaneous tracheostomy training.

**Level of evidence:**

Level III.

## Highlights

• The proposed training model for practice of surgeons in percutaneous tracheostomy is feasible.
• All responders concluded the training successfully.
• It is a possible alternative to percutaneous tracheostomy training.

## Introduction

Tracheostomy is a common procedure performed in patients with airway problems, especially in those with indication for prolonged orotracheal intubation. Because of the improved safety of the airways in tracheostomized patients, they present greater mobility practicality and better chances to receive physiotherapy. In addition, tracheostomy provides enhanced comfort to patients because it enables speech and oral feeding. Furthermore, weaning in tracheostomized patients is easier, because the ventilation tube can be easily reconnected in these patients, unlike in those submitted to orotracheal ventilation, who need to be sedated and reintubated in case weaning is not well supported.^1^

Traditionally, tracheostomy is a procedure performed in a surgery room. Percutaneous Dilatation Tracheostomy (PDT), which is routinely performed with the aid of bronchoscopy, was first described in 1985. In recent years, the original technique created by Ciaglia for percutaneous tracheostomy has undergone modifications, and several other procedures have been developed.^2^ Tracheostomy is a process that involves creating an opening in the anterior tracheal wall. Surgical tracheostomy consists in placing a tracheostomy cannula under direct vision after dissection of the pretracheal tissues and incision of the tracheal wall. PDT consists of blunt dissection of the pretracheal tissues, followed by dilatation of the trachea over the guidewire and insertion of a tracheal cannula using the Seldinger technique.^3^, ^4^

PDT can also be performed at bedside, thus escaping high-risk transfers of patients in severe conditions to operation room, in addition to being cost-effective. Therefore, costs associated with PDT are usually lower than those associated with surgical tracheostomy. Moreover, the time demanded for this procedure is usually shorter, reflecting its technical ease, and it is also normally associated with less blood loss and lower infection rates. These findings indicate the existence of a minimal dead space separating the tracheostomy tube and adjacent pretracheal tissues after PDT, which may cause a compression effect on minor bleeding, serving as a barrier to infection.^1^

Considering that the PDT technique requires a learning curve to be performed in the safest way possible for the patient, it is interesting to provide courses or practical classes that teach and offer practice of this technique. However, data on how the learning curve of this process occurs are scarce in the medical literature, and there are few studies demonstrating practical and accessible techniques for learning the procedure before it is performed in patients. Because there are not many studies addressing practical models in the literature, our proposal is to introduce a new type of mixed model, both synthetic and biological, that can train surgeons well in a context of greater resemblance to consistency and depth of the anatomic structures topographically anterior to the upper airway of patients.

Propose a model for performing percutaneous tracheostomy training using accessible material that enables comprehensive learning of the technique.

## Methods

The model comprises a structure composed of a wood board resting on a PVC tube. A foam block on which the pig trachea is supported is placed on this structure. A foam sheet covered with fabric is placed over the trachea to simulate the soft parts of the neck and the skin.

The wood board used is actually a clipboard from which the clip was removed. It is a 24.8 × 34 cm, Eucatex manufactured board. Four holes, vertically arranged two by two, were made at the top of the clipboard using a drill. At the same distance, four holes were drilled in a 5 cm-diameter, 25 cm-long, PVC tube in order to fix it to the clipboard. The pieces were joined using two 2.5 × 150 mm, black, nylon clamps. The purpose of fixing the PVC tube to the wood board is to give the structure a certain slope, and not leaving it totally horizontal on the table, providing closer resemblance to reality. A 24 × 5 × 7 cm, gray, D45 density foam block was glued on the wood board. Using a penknife, a 2 cm-deep central keel, approximately 8 cm-wide at the top, tapering to 2.5 cm in width after 8 cm down the keel was made on this foam block. Two 2 cm-wide, 27.5 cm-long, self-adhesive contact, Velcro fasteners were stuck on both sides of the foam block. The other parts of the Velcro fasteners were stuck to a 24 × 34 × 1.5 cm, white, D16 density foam sheet. This way, the foam block and sheet could be fastened to each other. A 24 × 34 cm piece of dull nylon fabric (satin and foam coupled layers) was glued on the white foam sheet.

A pig trachea was placed on the keel of the gray foam block and fixed with two needles at its lower and upper ends so that it remained stretched to facilitate palpation, because the pig trachea has spaces between the tracheal rings smaller than those of the human respiratory tract.

Preparation of the model is aimed at the practice of surgeons in percutaneous tracheostomy. The application of a questionnaire on the assessment of the proposed model to each participating surgeon is part of the methodology, as hereinafter described.

Ahead we present the step-by-step construction of the model (Figs. 1‒21) listing each material used for its preparation, the phases of percutaneous tracheostomy - in photographs taken of a surgeon applying the procedure at the Department of Surgical Technique of the “Irmandade da Santa Casa de Misericórdia de São Paulo”, and the responses to the questionnaire applied to the physicians after they had undergone training on the procedure using the experimental model.

**Fig. 1:**
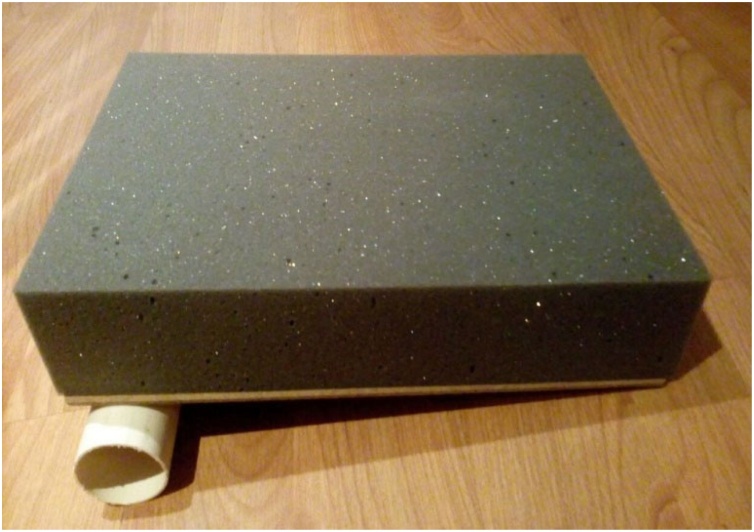
Wood board attached to the PVC tube with foam block on top (before the keel was cut).

**Fig. 2:**
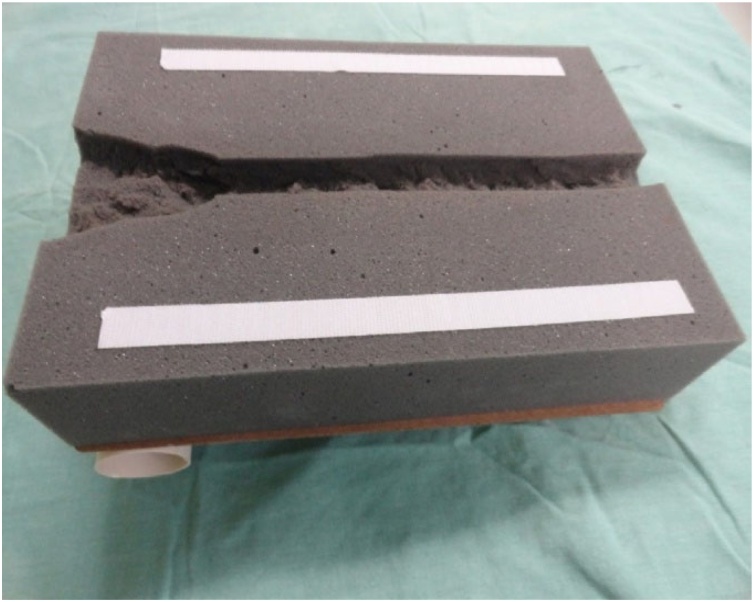
Part of the model inferior to the pig trachea with the keel and Velcro fasteners.

**Fig. 3:**
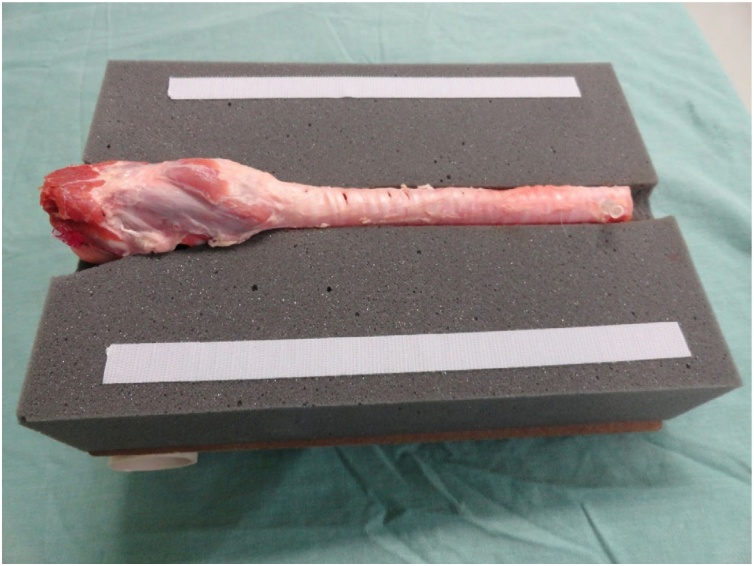
Model with pig trachea without superior foam sheet.

**Fig. 4:**
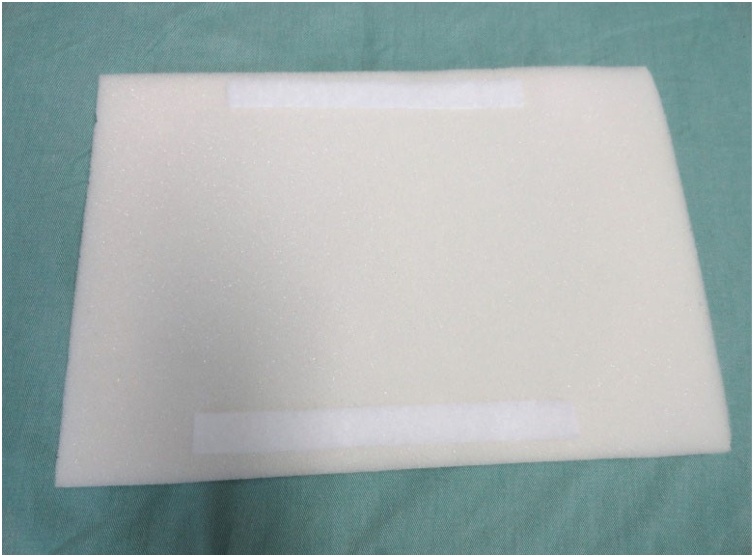
Bottom side of superior foam sheet with Velcro fasteners.

**Fig. 5:**
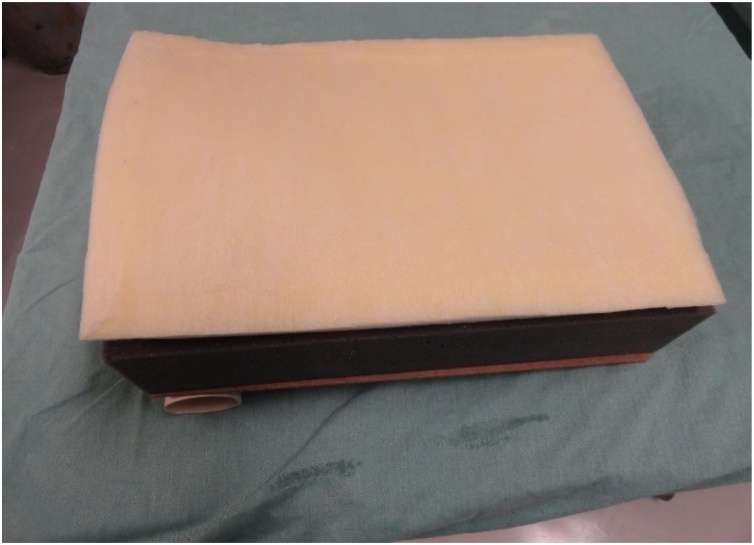
Complete assembled model.

**Fig. 6:**
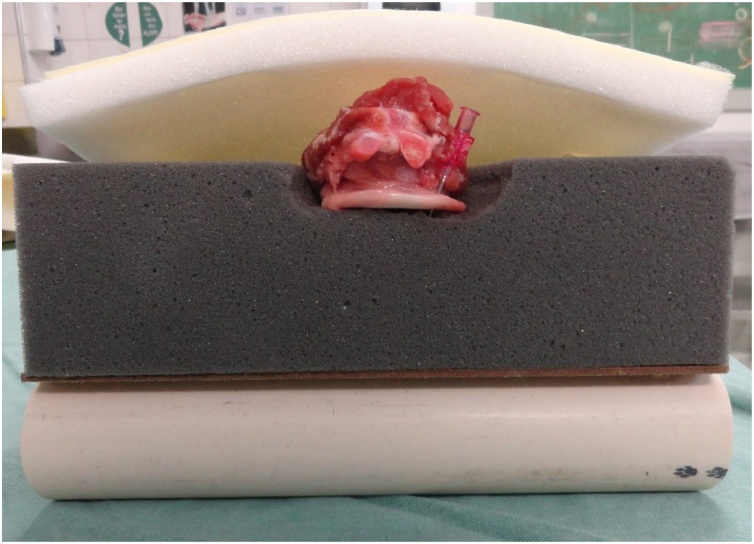
Superior part of the pig trachea fixed with a needle.

**Fig. 7:**
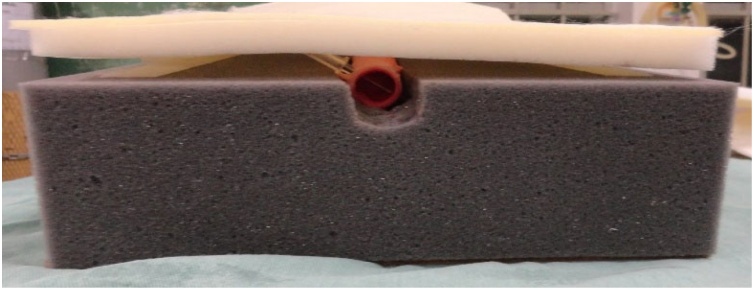
Inferior part of the pig trachea fixed with a needle.

**Fig. 8:**
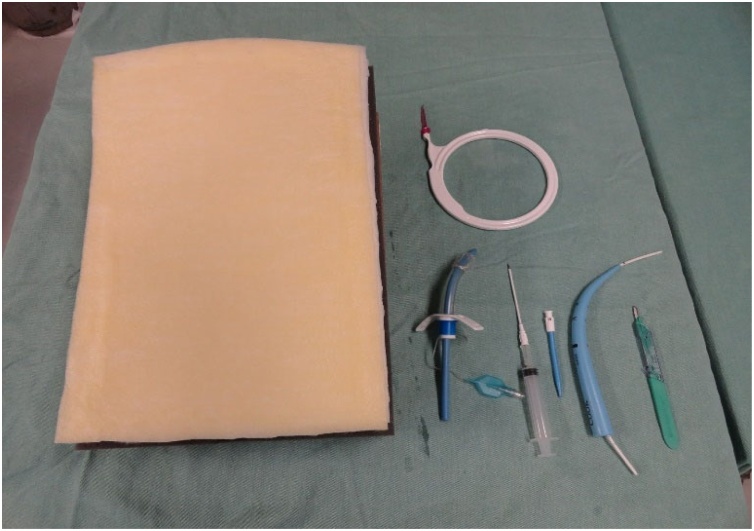
Model and material for performing the tracheostomy.

**Fig. 9:**
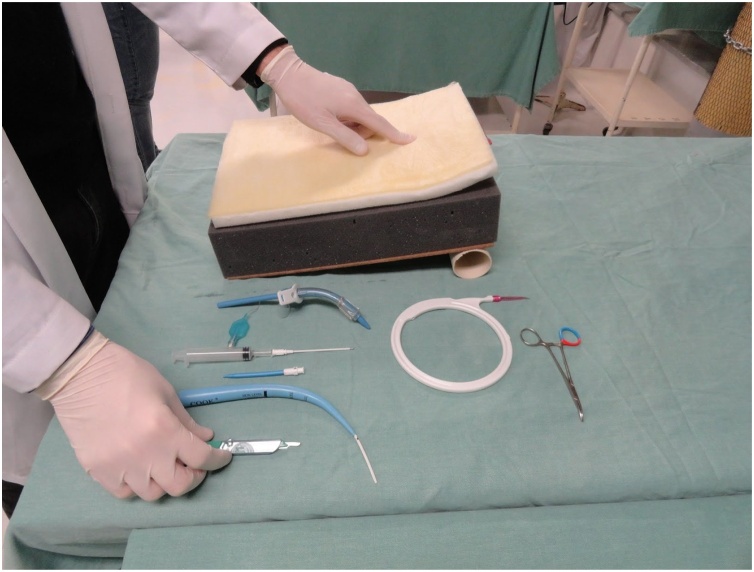
Beginning of the procedure. Palpation of the pig trachea.

**Fig. 10:**
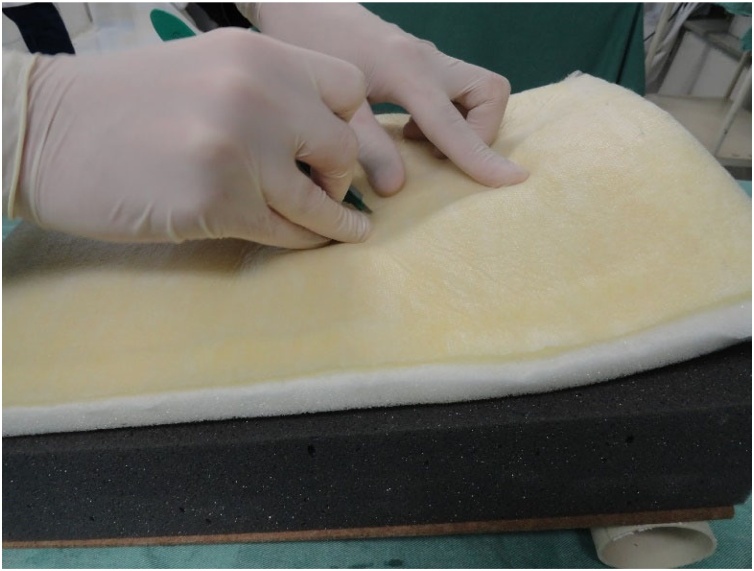
Initial incision in the fabric that simulates the skin.

**Fig. 11:**
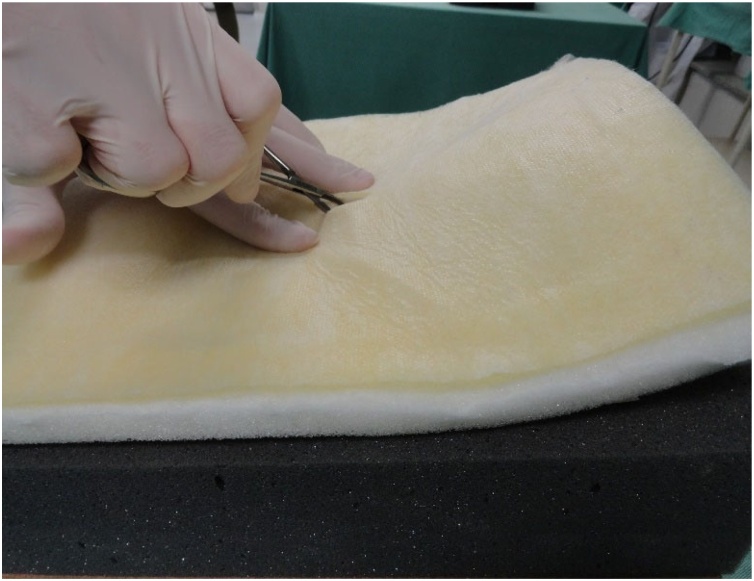
Divulsion of the foam that simulates the subcutaneous tissue.

**Fig. 12:**
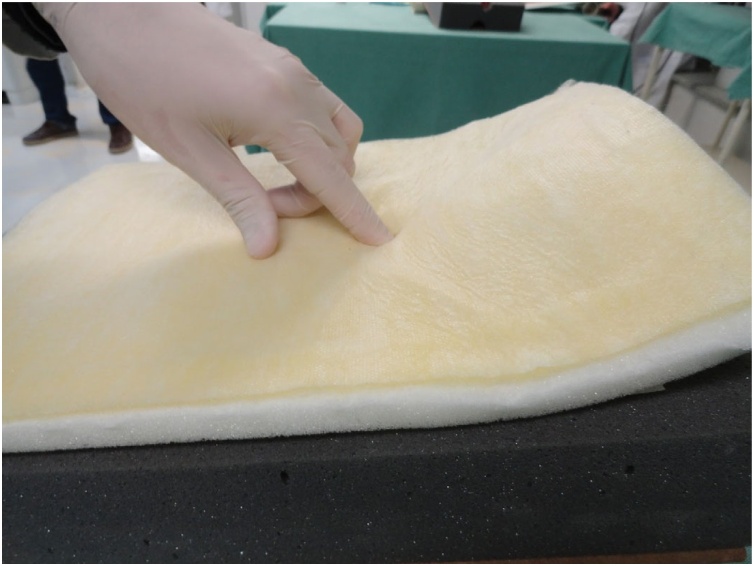
Palpation of the pig trachea through the opening.

**Fig. 13:**
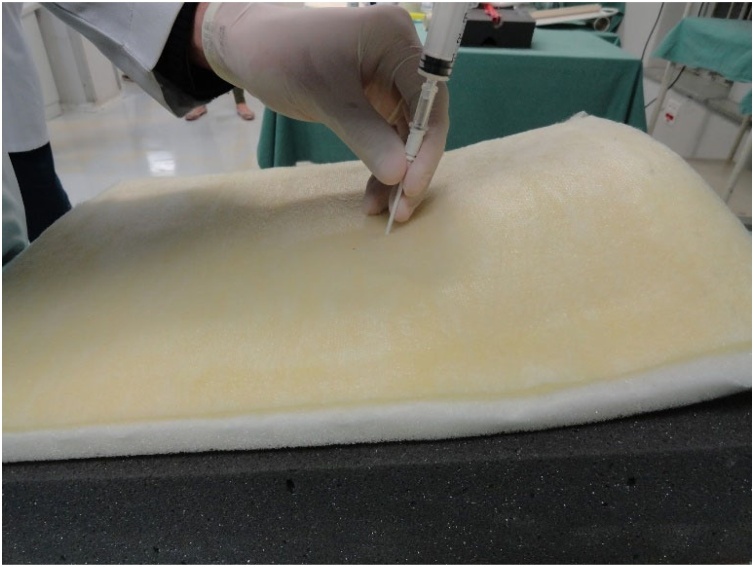
Passage of the needle through the pig trachea.

**Fig. 14:**
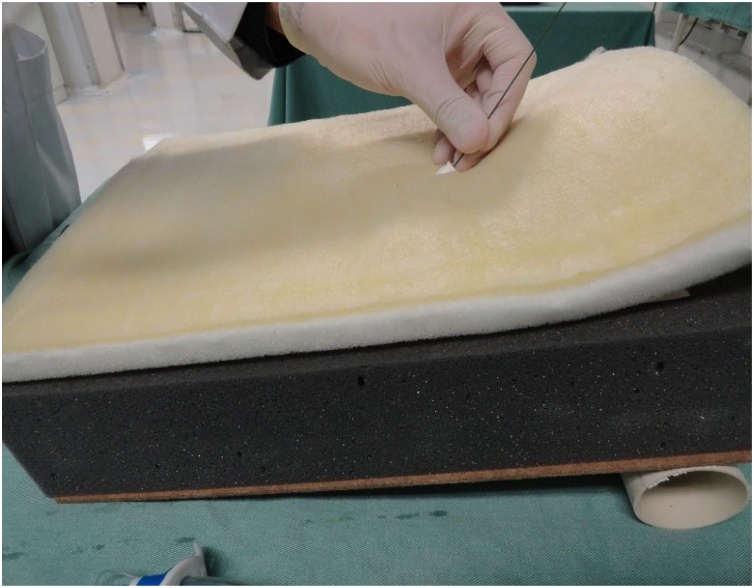
Passage of the guidewire.

**Fig. 15:**
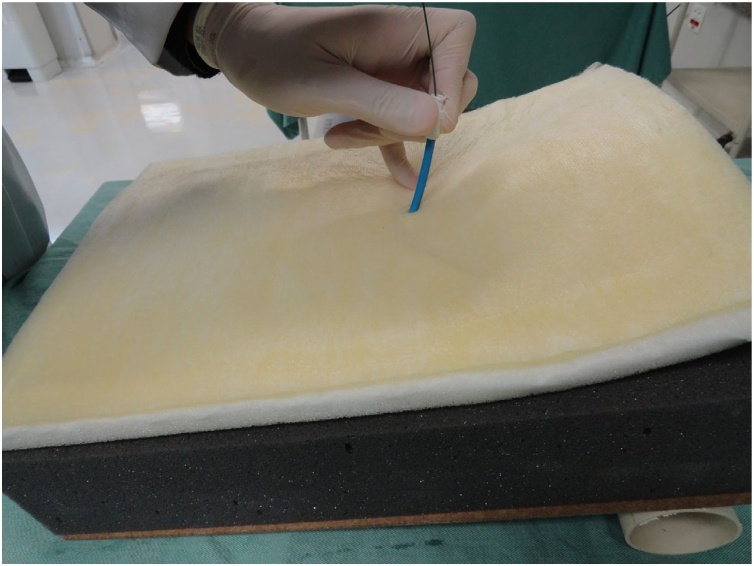
Dilation of the opening in the pig trachea.

**Fig. 16:**
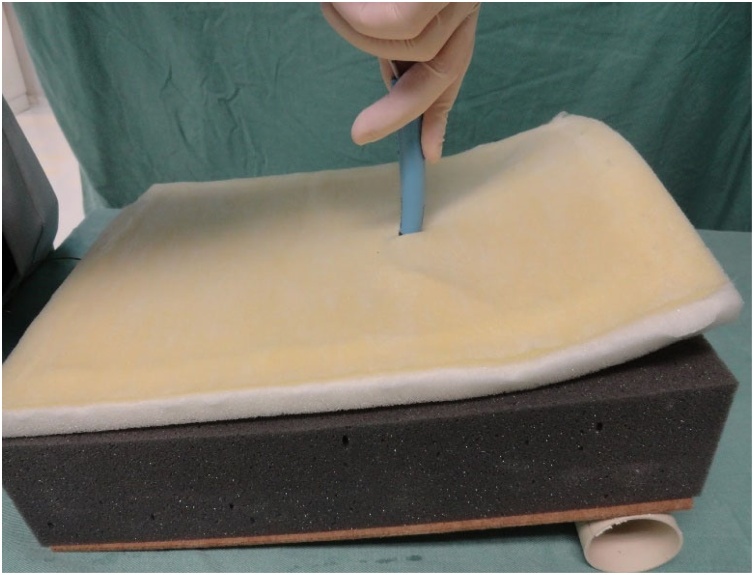
Dilatation.

**Fig. 17:**
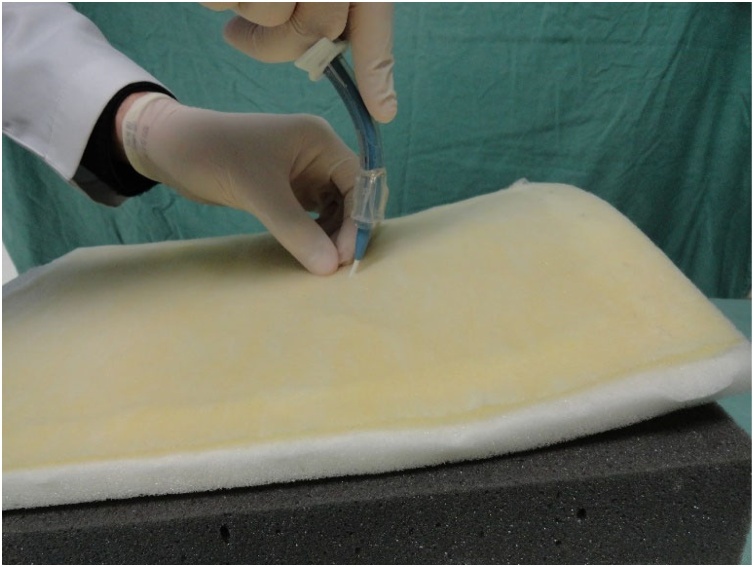
Guide with the cannula to be inserted into the pig trachea.

**Fig. 18:**
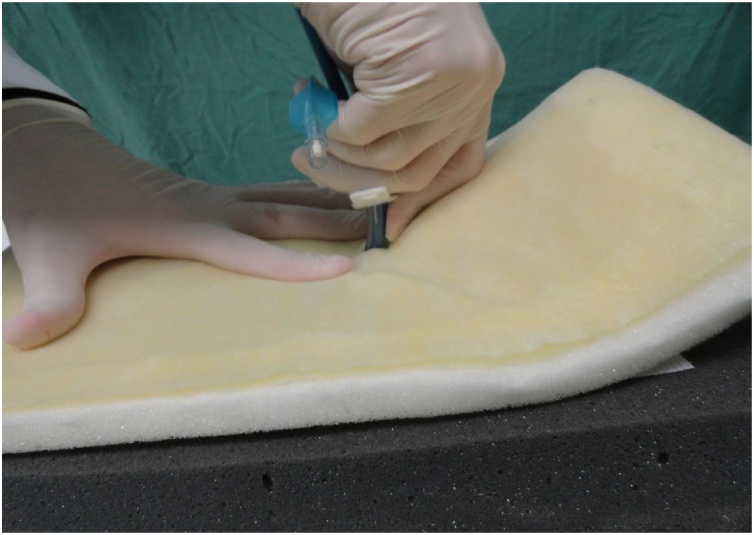
Insertion of the cannula.

**Fig. 19:**
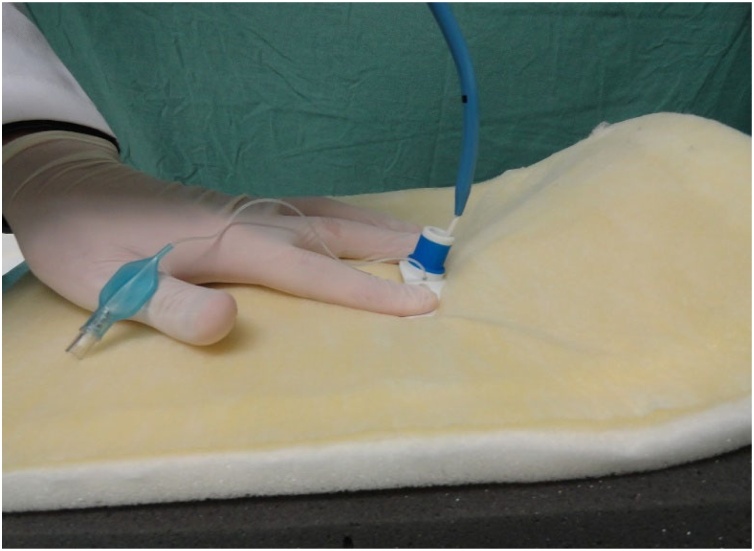
Removal of the guide and guidewire.

**Fig. 20:**
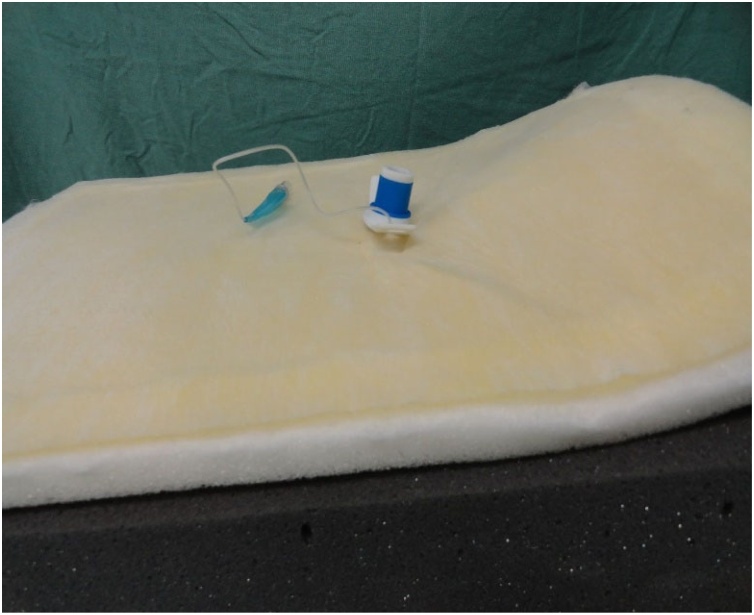
Cannula inserted in the model at the end of the procedure.

**Fig. 21:**
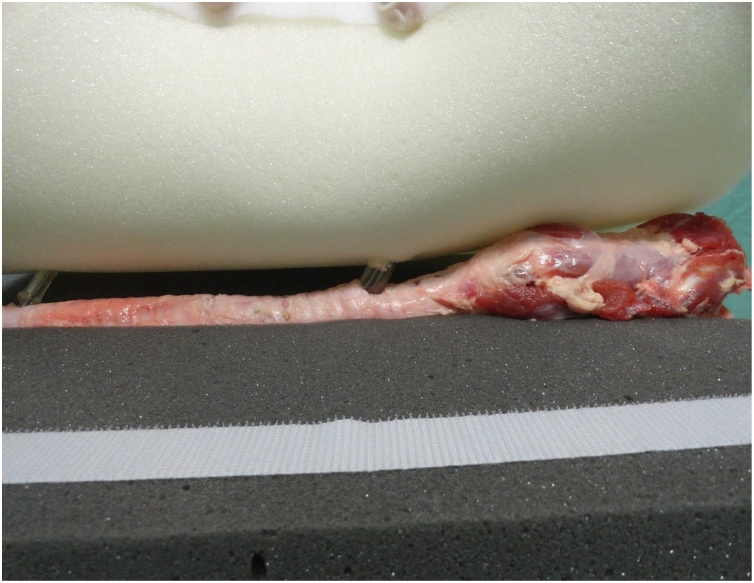
The Velcro fasteners allow the superior foam sheet to be lifted so that the success of the procedure can be verified.

### Procedure

The production cost per model is approximately R$ 52.00 (US$15.00), considering that the PVC tube, clamps, Velcro fastener, and dull fabric can be used to make more than one model (Table 1).

**Table 1:** Cost of the materials.

Materials	Price (R$)
Clipborad	2
PVC tube	4.5
Nylon clamp	5
Gray foam block	25
Pig trachea	10
White foam sheet	0.4
Velcro fastener (90 cm)	22.1
Dull fabric (1 × 1 m)	6
Total	75

## Results

Ten surgeons underwent training on the model: two experienced head and neck surgeons, three specialty residents, one otorhinolaryngologist, and three general surgery residents. All of them were able to perform the procedure successfully.

A questionnaire composed of six questions (Table 2) was applied to the participating physicians, in which they attributed a score ranging from one to five to each question, with five as the best score and one as the worst. The first question referred to the overall score of the model, to which six surgeons attributed a scored of five (very good) and three a scores of four (good). As for resemblance of the model to reality, seven physicians attributed it a score of four (very similar), one a score of three (similar), and one a score of two (little similar). When asked whether the model could be applied to training and learning, seven of the participants gave it a score of five (yes, certainly) and two a scores of four (yes, but it needs improvement). With respect to texture, four physicians attributed it a score of four (very similar) and five a scores of three (similar). Regarding resistance, one participant gave it a score of five (identical to reality), five a scores of four (very similar), two a scores of three (similar), and one a score of two (little similar). Concerning appearance, three surgeons attributed it a score of five (excellent), four a scores of four (very good), and two a scores of three (good, but it needs improvement).

**Table 2:** Results of the questionnaire.

Questions	Scores				
	5	4	3	2	1
Model general score	6	3	0	0	0
Resemblance to reality	0	7	1	1	0
Application in training and learning	7	2	0	0	0
Texture	0	4	5	0	0
Resistance	1	5	2	1	0
Appearance	3	4	2	0	0

## Discussion

Percutaneous Dilatation Tracheostomy (PDT), as reported in previous studies, presents a learning curve that was more prominent in the first 20 patients submitted to the procedure. Most complications occur when apprentices are still gaining experience in performing the procedure; therefore, it should always be performed in the presence of an experienced surgeon. After adequate experience to perform PDT has been acquired, the procedure performed at patient’s bedside can be safe and efficient.^5^

Considering the PDT learning curve, it is interesting to propose training environments for the technique before it is performed in patients. The literature presents some cohort studies conducted to evaluate the learning curve of PDT; however, they assess procedures performed in patients, rather than in experimental models.^6^

Training models previously described include medical mannequins or animals such as sheep; however, they present high-cost effectiveness, hindering access and performance.^7^

Medical mannequins for performing procedures are an alternative available in the market, but they are expensive. Options of homemade models are an interesting alternative to gain familiarity with the procedure, and are much more affordable with regard to their cost; however, they may bear little resemblance to real tissue conditions. Animals end up being an effective, not so expensive option that tends to present closer resemblance to the real texture of biological tissues.^7^

## Conclusion

The experimental model constructed has met its purpose, and its low production cost and verisimilitude to the texture of the human cervical region demonstrate that the model can be a new efficient and inexpensive alternative to be used in teaching hospitals.

The proposed model is especially important with regard to teaching hospitals and the public health system, which often present financial limitations. Therefore, it enables courses and test practices to be performed accessibly, especially with resident physicians, assisting with the learning curve of percutaneous dilatation tracheostomy PDT and enabling it to be more safely performed in patients who need it.

Further studies are needed to verify the quality of the proposed model and its resemblance to biological tissues. Future testing of the model performed with participation of head and neck and general surgeons, their assessment of the model, and comparison with surgical practice, are an alternative of continuity study to better evaluate the proposed experimental model.

## CRediT authorship contribution statement

CSN and KSS ‒ Medical Students, Faculdade de Ciências Médicas da Santa Casa de São Paulo (FCMSCSP). MYN ‒ MD, Head and Neck Surgeon; Physician of Improvement Course, Head and Neck Surgery of the Irmandade da Santa Casa de São Paulo. ABS ‒ MD, MSc; Assitant, Head and Neck Department, Irmandade da Santa Casa de Misericórdia de São Paulo. AJG ‒ Associate Professor; Chief, Head and Neck Department, Irmandade da Santa Casa de Misericórdia de São Paulo.

## Declaration of competing interest

The authors declare no conflicts of interest.
